# PIP4K2A/2B inhibitor suppresses tumor growth in a xenograft model of NSCLC

**DOI:** 10.1016/j.isci.2026.115952

**Published:** 2026-04-30

**Authors:** Zunyu He, Song Chen, Marcus Bosenberg, Viswanathan Muthusamy, Yibo Xi, He Wang, Fabrizio Micheli, Agostino Cianciulli, Claudia Beato, Michael Van Zandt, Jonathan Ellman, Ya Ha

**Affiliations:** 1 Department of Pharmacology, Yale School of Medicine, New Haven, CT 06520, USA; 2 Yale Center for Precision Cancer Modeling, Yale School of Medicine, New Haven, CT 06520, USA; 3 Department of Laboratory Medicine and Pathology, Yale School of Medicine, New Haven, CT 06520, USA; 4 Aptuit Srl, an Evotec Company, Via Fleming 4, 37135 Verona, Italy; 5 New England Discovery Partners, Branford, CT 06405, USA; 6 Department of Chemistry, Yale University, New Haven, CT 06520, USA

**Keywords:** Pharmacology, Cancer

## Abstract

The PIP4K family of lipid kinases phosphorylates the rare phospholipid PI(5)P at the 4-position, generating PI(4,5)P_2_ inside the cell. Although the functions of PIP4K, as well as the intracellular pools of PI(5)P and PI(4,5)P_2_, remain incompletely understood, there are emerging interests in developing inhibitors to target these kinases since their genetic ablations have broad tumor-suppressive and other beneficial effects. Here, we report continued optimization of a previously discovered 2-amino-dihydropteridinone PIP4K2A/2B inhibitor and demonstrate, for the first time that pharmacological inhibition of PIP4K2A/2B suppresses solid tumor growth *in vivo*. The tumor-suppressive effect of the inhibitor appears to be non-tumor cell-autonomous and is likely mediated by components of the tumor microenvironment, including macrophages that commonly adopt alternatively activated states and support tumor growth. Our findings suggest a potential role for PIP4K activity in tumor-associated macrophages and provide a rationale for further exploring pharmacological targeting of these lipid kinases in cancer.

## Highlights

• Pharmacological inhibition of PIP4K suppresses NSCLC tumor growth *in vivo*
• Tumor suppression is driven by non-tumor cell-autonomous mechanisms
• PIP4K in tumor-associated macrophages represents a therapeutic target

## Graphical abstract

**Figure:**
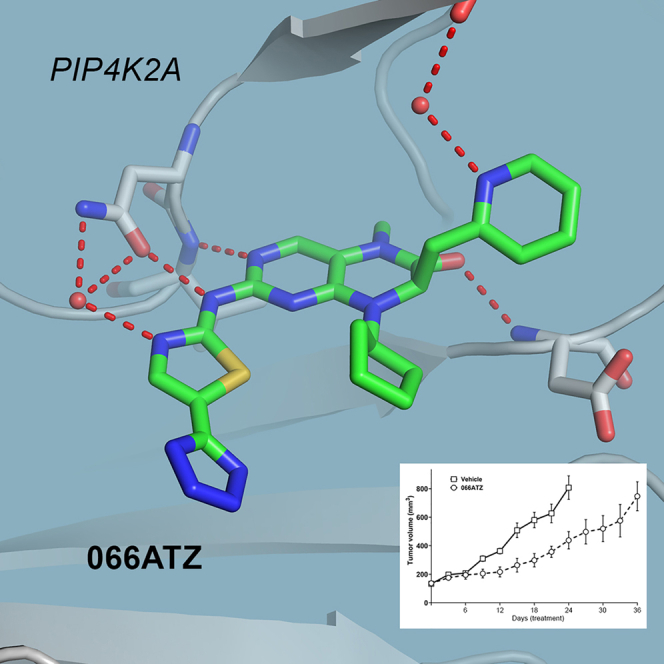

## Introduction

The PIP4K family of lipid kinases is abundantly expressed by all multicellular eukaryotic organisms.^1^^,^^2^^,^^3^^,^^4^ They phosphorylate the 4-OH of phosphatidylinositol 5-phosphoate (PI(5)P), converting the lipid to phosphatidylinositol 4,5-bisphosphate (PI(4,5)P_2_).^5^^,^^6^ The biological functions of these kinases are, however, not fully understood as most PI(4,5)P_2_ in a cell is produced by the related PIP5K family of lipid kinases.^7^^,^^8^ In mammals, the PIP4K family has three isoforms (α, β, and γ) that are encoded by *PIP4K2A*, *PIP4K2B*, and *PIP4K2C*, respectively. Among the three, the α and β isoforms are significantly more active, at least when assayed for enzymatic activity *in vitro*.^9^ In cells where the kinases’ subcellular location has been examined, the α isoform is predominantly cytosolic whereas the β isoform is mostly found in the nucleus, suggesting that the two active kinases are likely involved in different processes.^10^^,^^11^^,^^12^^,^^13^ Nevertheless, the possibility that the three PIP4Ks could heterodimerize complicates these simple categorizations.^14^^,^^15^ The substrate for PIP4K, PI(5)P, is of extremely low abundance and has been associated with stress response.^16^^,^^17^^,^^18^ Therefore, one function of the PIP4Ks could simply be to remove PI(5)P, adding it to the more stable pool of PI(4,5)P_2_. More recently, several studies have uncovered previously unknown functions for PIP4K-mediated endomembrane PI(4,5)P_2_ that are different from those of plasma membrane PI(4,5)P_2_.^11^^,^^12^^,^^19^

There are substantial interests in developing pharmacological agents to target the PIP4Ks.^20^ The PIP4K2A knockout (KO) mice do not have any overt phenotype but knocking out PIP4K2B and PIP4K2C alter animal metabolism and immune response, respectively.^4^^,^^21^^,^^22^ Most strikingly, simultaneous ablation of α and β isoforms (PIP4K2A^−/−^PIP4K2B^+/−^) in TP53^−/−^ mice broadly protects the animals from spontaneous tumorigenesis caused by the inactivation of tumor suppressor p53.^4^ This latter finding raises hope that loss-of-function p53 mutations, commonly found in many human cancers and difficult to repair by conventional pharmacological means, could be targeted by PIP4K2A/2B dual inhibitors either prophylactically or therapeutically.^23^

The role of the PIP4Ks in cancer is probably complex and context dependent. The synthetic lethal interaction between PIP4K2A/2B and p53 observed in some tumor cells could relate to their overlapping functions in regulating cell metabolism and autophagy.^4^^,^^11^^,^^19^^,^^24^^,^^25^^,^^26^ This cell-autonomous mechanism was elegantly demonstrated *in vivo* by two sarcoma studies, one examining tumor initiation using KP mice (Kras^LSL−G12D/+^TP53^flx/flx^) and the second examining allograft tumor growth upon inducible PIP4K2A/2B knockdown.^19^ In acute myeloid leukemia (AML) cells, inactivation of PIP4K2A alone causes accumulation of CDK inhibitors CDKN1A and CDKN1B, which hampers cell survival and proliferation.^27^ Furthermore, inhibition of PIP4K2B and PIP4K2C selectively impacts the function of regulatory T cells (Tregs).^22^^,^^28^ Tregs play an important role in immuno-suppression within the tumor microenvironment (TME). Therefore, their inactivation is expected to have a large inhibitory effect on solid tumor growth in a non-cell-autonomous manner.

In this study we used an improved lipid kinase inhibitor, based on the previously reported 2-amino-dihydropteridinone core,^25^ to examine the effect of PIP4K inactivation on tumor growth using a xenograft model of lung adenocarcinoma (LUAD), a type of non-small cell lung cancer (NSCLC).^29^ LUAD is one of the late-onset tumors developed by TP53^+/−^ mice, resulting from loss-of-heterozygosity (LOH) mutations in TP53 within alveola cells.^30^^,^^31^ In contrast to our initial hypothesis that the PIP4Ks could be synthetic lethal with TP53 within LUAD cells, we found that PIP4K inhibitor suppressed xenograft tumor growth by altering the function of tumor associated macrophages (TAMs), which are major innate immune cells in the TME and usually promote tumor growth.^32^ This discovery reveals an additional mechanism by which PIP4K inhibition could be exploited for cancer treatment.

## Results

### PIP4K2A/2B inhibitor with improved metabolic stability

**Figure 1:**
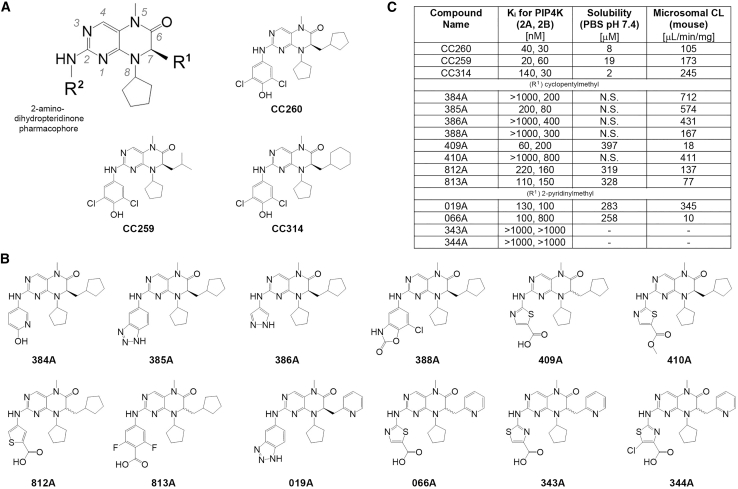
Improving mouse liver microsomal stability for the 2-amino-dihydropteridinone PIP4K2A/2B inhibitors (A) Three previously obtained and potent inhibitors share a dichlorophenol R2 side chain.^25^ The chiral center (C7) and the large hydrophobic R^1^ side chain confer lipid kinase specificity. (B) The dichlorophenol in CC260 was replaced by various chemical groups with at least one aromatic ring and a (partial) negative charge. Some compounds have a 2-pyridinylmethyl R^1^ side chain instead of CC260’s cyclopentylmethyl side chain. (C) The compounds’ solubility and mouse liver microsomal stability were compared. N.S., not soluble. The K_i_(s) for PIP4K2A and PIP4K2B were estimated from a single inhibitor (100 nM) and ATP (20 μM) concentration using the ^32^P-based TLC lipid kinase assay.^25^

We devoted substantial initial efforts to improving the lead 2-amino-dihydropteridinone derivatives’ metabolic stability by replacing a potentially labile R^2^ dichlorophenol side chain (Figures 1A and 1B).^25^ The dichlorophenol contributes to the compound’s potency by intercalating into a hydrophobic cleft adjacent to the lipid kinase’s ATP-binding site (between Phe-134 and Phe-200; PIP4K2A numbering) and by forming a salt bridge with the nearby Lys-209. To maintain these favorable interactions, several aromatic groups with an opposing acidic moiety were installed onto the 2-amino-hydropteridinone core. Most substitutions retained potency toward the lipid kinases but failed to improve, or even worsened, the compound’s liver microsomal stability (Figure 1C). Only two compounds, 409A and 066A, sharing a thiazole carboxylic acid group, demonstrated reasonable resistance to phase 1 metabolism by the liver enzymes.

**Figure 2:**
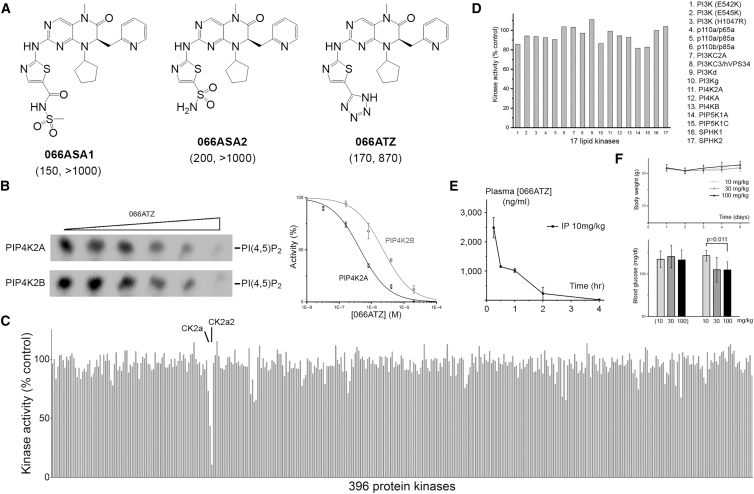
066ATZ maintains kinase selectivity and has improved *in vivo* half-life (A) The carboxylic acid moiety in 066A was replaced by various bioisosteres to reduce phase II metabolism. The K_i_(s) for PIP4K2A and PIP4K2B are shown in the parentheses (nM). (B) The concentration-inhibition curves for PIP4K2A and PIP4K2B (*n* = 3, mean ± SEM). One representative set of autoradiographs of the TLC plates over a range of 066ATZ concentrations (0, 0.03, 0.16, 0.8, 4, and 20 μM) are shown. [ATP] is fixed at 20 μM in these experiments. (C) 066ATZ (0.5 μM) was profiled against 396 human protein kinases in the presence of 10 μM ATP. Only two protein kinases were significantly inhibited (CK2a, 57%; CK2a2, 90%). (D) 066ATZ (0.5 μM) was profiled against 17 lipid kinases in the presence of 10 μM ATP. (E) The time course of plasma 066ATZ concentration after IP injection into male C57BL/6 mice. Data are presented as mean ± SEM (*n* = 3). Dose, 10 mg/kg; C_max_, 2,484 ng/mL (∼5.1 μM); t_1/2_, 0.5 h. (F) The effects of 066ATZ on the body weight and blood glucose levels (15 min and 30 min after IP injection) were tested in female nude mice. Each dose group (10, 30, and 100 mg/kg) contained 5 animals. Data are presented as mean ± SEM (*n* = 5). A significant drop in blood sugar was observed in the two higher dose groups 30 min post injection. *p* values were calculated using Student’s *t* test in GraphPad Prism 10.0 (GraphPad Software, San Diego, CA).

066A was selected for further optimization. The carboxylate functional group was replaced by an acylsulfonamide, sulfonamide, or tetrazole to improve membrane permeability and to overcome phase 2 metabolism (Figure 2A).^33^ The new derivatives were similarly active against PIP4K2A but lost some potency against PIP4K2B (Figure 2B). Profiling the tetrazole (066ATZ) against a panel of protein and lipid kinases demonstrated better selectivity than the earlier, albeit more potent, inhibitor CC260 that cross-reacted with PI3Kγ and PI3Kδ (Figure 1A).^25^ 066ATZ inhibited two closely related protein kinases, CK2a and CK2a2 (casein kinase 2 catalytic subunits α and α′; calculated K_i_ ∼130 nM and 40 nM, respectively). The two casein kinases are constitutively active. Their inhibition was unanticipated and remains unexplained because 066ATZ retains a large R^1^ side chain, which should sterically clash with a salt bridge conserved in all protein kinases.

066ATZ has a half-life of ∼0.5 h in mice (Figure 2E). Since it is well tolerated (e.g., not affecting body weight at a dose of 100 mg/kg), a plasma concentration of 20 μM can be achieved and maintained for over an hour after each IP injection (100 mg/kg), which should yield high occupancy at the targeted lipid kinases. While investigating the maximum tolerated dose (MTD), we observed a transient 14% blood sugar drop 30 min after IP injection in the 30 mg/kg and 100 mg/kg groups but not in the 10 mg/kg group (Figure 2F). This response appears consistent with the genetic studies showing that PIP4K2A/2B knockout alters whole body metabolism.^4^^,^^21^

**Figure 3:**
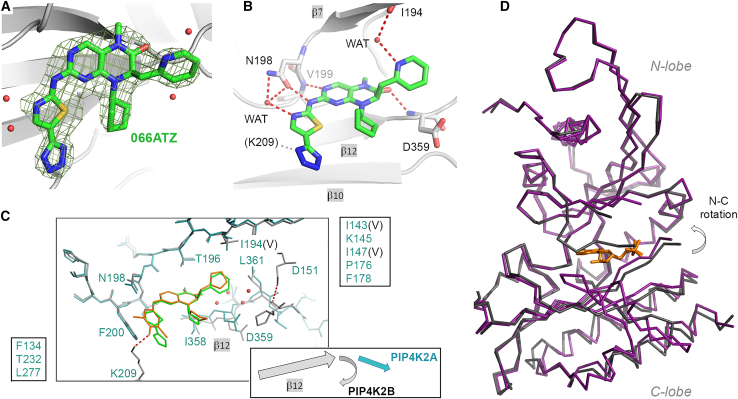
The crystal structure of 066ATZ in complex with PIP4K2A (A) Fo-Fc map, contoured at 3.0 sigma, confirms the presence of the bound inhibitor. The apo PIP4K2A structure (PDB: 7N6Z),^25^ with solvents and ions removed, was used in rigid body refinement and phase calculation. (B) The binding mode of 066ATZ is identical to those of other 2-amino-dihydropteridinone inhibitors. The newly installed 2-methylpyridine forms a water-mediated hydrogen bond with the backbone carbonyl of Ile-194. The nitrogen in the thiazole ring forms water-mediated hydrogen bonds with the side chain of Asn-198. Although disordered in the crystal, the side chain of Lys-209 is predicted to form a salt bridge with N1 of the tetrazole group. (C) The PIP4K2A/066ATZ (blue and green) and PIP4K2B/CC260 (gray and orange; PDB: 7N81) structures were superimposed based on the proteins’ N-lobe backbone atoms only. PIP4K2B has a slightly wider gap between the N- and C-lobes, as illustrated by the shift of the peptide segment between Ile-358 and Leu-361 (PIP4K2A numbering). Protein residues in contact with the bound inhibitors were labeled (PIP4K2B in black; those omitted for clarity were listed in the boxes on either side of the diagram). The protein backbone in all experimentally determined PIP4K2B structures has a sharp bend immediately following Leu-361, which is absent in PIP4K2A. (D) PIP4K2B demonstrates some interdomain conformational flexibility. In the diagram, the N-lobes of apo (gray; PDB: 7N80) and AMPPNP-bound (purple; PDB: 6K4H) PIP4K2Bs were superimposed, revealing a slight movement in the C-lobe.^25^^,^^41^ None of the solved PIP4K2A structures has this flexibility.

Given genetic evidence implicating the PIP4Ks in metabolic regulation, cancer and other pathological conditions, multiple research groups have attempted to develop potent and selective PIP4K inhibitors.^24^^,^^25^^,^^34^^,^^35^^,^^36^^,^^37^^,^^38^^,^^39^^,^^40^ Intriguingly, most reported compounds show better activity toward PIP4K2A than PIP4K2B, in agreement with our experience. To help explain why potent PIP4K2A inhibitors are often less effective against PIP4K2B, we solved the crystal structure of 066ATZ in complex with PIP4K2A (Table S1, Figures 3A and 3B), and compared it to that of CC260 bound to PIP4K2B (Figure 3C). Within the two complexes, the 2-amino-dihydropteridinone core is bound identically. Most protein side chains in contact with the compounds are also identical. Nevertheless, we noted three subtle differences. (1) The N-lobe side (“ceiling”) of the inhibitor-binding pocket harbors three conservative substitutions (I143(V), I147(V), I194(V); PIP4K2B residues in parentheses), which make PIP4K2B’s binding pocket slightly wider. (2) In PIP4K2B, the β-strand (β12) that forms the “floor” of the pocket adopts a sharp bend immediately following Leu-361 (PIP4K2A numbering). The bend does not appear to be an artifact of crystallization since it has been observed in every PIP4K2B structure from the PDB,^25^^,^^41^^,^^42^^,^^43^ thus distinguishing PIP4K2B from other experimentally solved or AlphaFold-predicted PIPK structures. The bend is stabilized by Asp-151 and several water molecules (red spheres in Figure 3C). Ligand binding could perturb this water network. (3) Unlike PIP4K2A, PIP4K2B possesses a small degree of rotational flexibility between the N- and C-lobes (Figure 3D), which also affects the width of the ligand binding pocket. The slightly closed structure, e.g., the one with a bound AMP-PNP,^41^ superposes better with that of PIP4K2A.

### 066ATZ suppresses solid tumor growth

**Figure 4:**
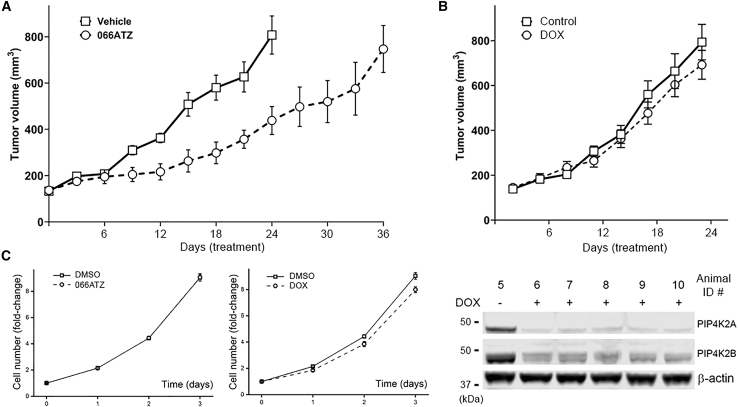
066ATZ reduces tumor growth rate in a xenograft model of NSCLC (A) The growth curves of H1975∗ xenograft tumor in R2G2 mice. Data are presented as mean ± SEM (*n* = 5). 066ATZ (100 mg/kg) or vehicle was IP injected 5 times a week after the tumors reached ∼150 mm^3^. One animal died in the treatment group on day 39 for an unknown cause when the tumor reached a size of ∼550 mm^3^. All other mice were sacrificed when the tumors reached ∼1,000 mm^3^. On day 24, the average tumor volume was significantly smaller in the treatment group (*p* < 0.01). *p* values were calculated using Student’s *t* test (two-tailed unpaired *t* test) with Welch’s correction in GraphPad Prism 10.0 (GraphPad Software, San Diego, CA). (B) The growth curves of H1975∗ xenograft tumor in R2G2 mice after DOX-induced PIP4K2A/2B knockdown. Data are presented as mean ± SEM (*n* = 5). When the tumors reached ∼150 mm^3^, DOX was administered to the mice continuously through drinking water (1 mg/mL). Tumor tissues were harvested at the end of the experiment, homogenized, and analyzed by WB (shown below the growth curves). (C) *In vitro*, neither 066ATZ (20 μM) nor DOX (0.2 μg/mL) affected the growth rate of cultured H1975∗ cells. Data are presented as mean ± SEM (*n* = 6). The cell numbers were measured by CellTiter-Glo.

The improved pharmacokinetics enabled us to study, for the first time, the effect of PIP4K2A/2B inhibitor on solid tumor growth *in vivo*. We tested 066ATZ in a xenograft model of NSCLC, using an engineered H1975∗ cell line expressing the tetracycline-inducible shRNA targeting PIP4K2A/2B. IP injection of 066ATZ five times a week significantly reduced tumor growth (Figures 4A and S1). In contrast, feeding the mice with doxycycline (DOX) through drinking water, which induced knockdown of PIP4K2A/2B only in H1975∗ cells, did not affect the tumor’s growth rate (Figure 4B). The possibility that PIP4K2A/2B inactivation within tumor cells might not have any direct impact on cell proliferation is further supported by the observation that, *in vitro*, the growth rate of cultured H1975∗ cells was not affected by 066ATZ treatment, or by DOX-induced knockdown of PIP4K2A/2B (Figure 4C; DOX itself does not affect H1975 cell growth). To rule out the possibility that off-target inhibition of protein kinase CK2α or CK2α′ by 066ATZ could contribute to the anti-tumor activity, mice were also treated with CX-4945, a sub-nanomolar CK2α/α′ inhibitor,^44^ which showed no effect on xenograft tumor growth (Figure S2).

**Figure 5:**
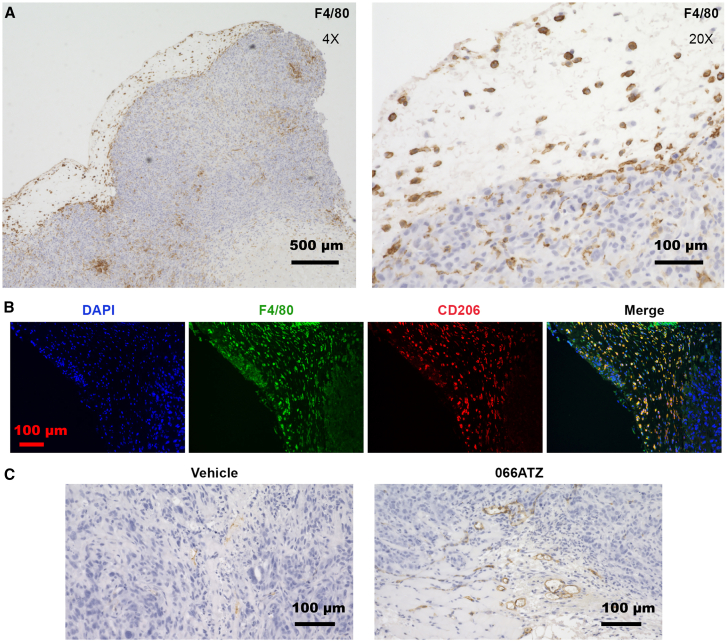
Representative IHC and IF images of H1975∗ xenograft tumor (A) IHC staining of tumor tissue (animal #2 in the vehicle group) with F4/80 antibody and hematoxylin. In all animals, the tumor cells (blue) are surrounded by a dense layer of murine F4/80+ macrophages (brown). In some animals (regardless of treatment), macrophages are also found in dense clusters within the tumor. Nevertheless, there is no correlation between macrophage infiltration and tumor growth rate. Scale bars, 500 (4× panel) or 100 μm (20× panel). (B) IF staining reveals that most F4/80-stained cells (green) are also positive for CD206 (red), a marker highly expressed by M2a and M2c macrophages. Scale bars, 100 μm. (C) IHC staining with CD31 antibody reveals that 066ATZ treatment is associated with enhanced angiogenesis (animal #1 in the vehicle group and animal #10 in the treatment group are shown). Scale bars, 100 μm.

It is conceivable that 066ATZ’s tumor-suppressive effect could be related to its ability to alter systemic metabolism. However, the degree by which host metabolic perturbation contributes to reduced tumor growth in this case remains uncertain since the drop in blood glucose concentration after 066ATZ injection was only transient, and there was no difference in steady-state sugar level after 4 consecutive daily treatments (Figure 2F). Histological examination of harvested tumor tissues did not reveal any obvious difference between 066ATZ and vehicle groups, except for a small increase in CD31 staining within compound-treated tumors (Figure 5C), suggesting that 066ATZ, counterintuitively, promoted angiogenesis instead of inhibiting it. Although the Rag2/IL2RG double knockout mice (R2G2) used in our xenograft experiment are deficient in T cells, B cells, and NK cells, and have reduced macrophages, neutrophils, and dendritic cells, immunohistochemistry (IHC) staining revealed that, in both compound-treated and control tumor tissues, dense populations of macrophage cells were recruited to the periphery of the xenograft tumor and, in some cases, penetrated deep into the tumor (Figures 5A and 5B). Since TAMs generally promote tumor growth,^45^^,^^46^ we investigated next whether PIP4K2A/2B inhibition could have impacted macrophage function.

### Inactivation of PIP4K2A/2B reduces M2a macrophage’s growth promoting activity

**Figure 6:**
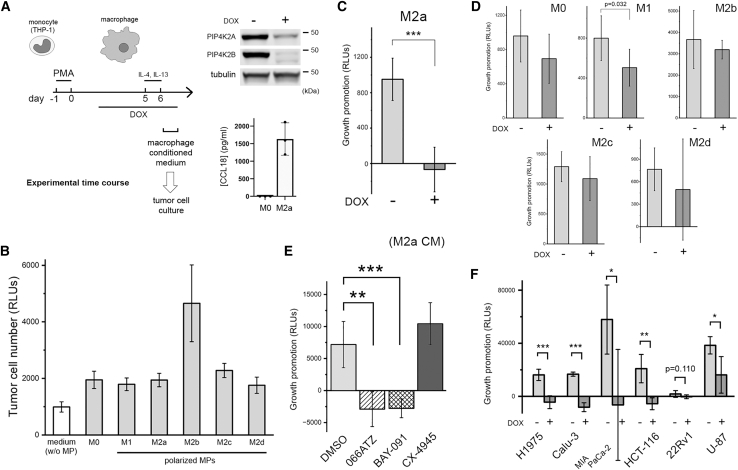
PIP4K2A/2B inactivation reduces macrophage’s activity in promoting tumor cell growth (A) A schematic diagram illustrating the time course of macrophage (MP) differentiation and polarization *in vitro*.^47^^,^^52^ THP-1∗ cells were treated with 200 nM PMA for 24 h to induce differentiation. The adherent cells were collected and replated (∼2,000,000 cells per well) on day 2. In the knockdown experiment, DOX (200 ng/mL) was added on day 3 and maintained throughout the experiment. In the compound treatment experiment, 066ATZ (20 μM) was added to the macrophage culture on day 5. Neither DOX nor 066ATZ affected macrophage viability. The following reagents were added on day 6 to induce macrophage polarization (only M2a polarization was shown in the diagram): M1, IFNγ (100 units/ml) and LPS (100 ng/mL); M2a, IL-4 (10 units/ml) and LPS (100 ng/mL); M2b, IgG-OVA complex and LPS (100 ng/mL); M2c, IL-10 (10 ng/mL); M2d, NECA (5 μM) and LPS (100 ng/mL). After removing polarization solutions on day 7, serum-free DMEM/F12 medium was added, and culture supernatant was collected after 24 h. The WB shown next to the time course diagram confirms DOX-inducible knockdown of PIP4K2A and PIP4K2B in THP-1∗ macrophages. M2a polarization caused an increase of CCL18 secretion, which was detected by ELISA. Data are presented as mean ± SEM (*n* = 3). (B) The conditioned media from unpolarized macrophages (M0) and differently polarized macrophages (M1, M2a-d) promoted H1975 cell growth. Data are presented as mean ± SEM (*n* = 6). The cell numbers were determined by CellTiter-Glo. The control medium was collected from a well without macrophages. During the 3 days growth period, H1975 cell number doubled in the control medium. To account for this residual growth, the difference in final cell numbers was used to quantify the growth promoting effect of macrophage conditioned medium. (C) Knockdown of PIP4K2A/2B in M2a polarized macrophage eliminated its growth promoting activity (∗∗∗, *p* < 0.0001). Data are presented as mean ± SEM (*n* = 6). Neither DOX-induced knockdown nor 066ATZ affected macrophage viability. *p* values were calculated using Student’s *t* test in GraphPad Prism 10.0 (GraphPad Software, San Diego, CA). (D) The effect of PIP4K2A/2B knockdown in other macrophages did not reach statistical significance, although in M1 polarized macrophages the knockdown appeared to produce a small reduction. Data are presented as mean ± SEM (*n* = 6). (E) Treatment with 066ATZ (20 μM), or with a more potent PIP4K inhibitor, BAY-091 (2 μM),^36^ reduced M2a macrophage’s growth promoting activity (∗∗, *p* < 0.001; ∗∗∗, *p* < 0.0001). Data are presented as mean ± SEM (*n* = 6). CK2α/α′ inhibitor CX-4945 (0.5 μM) had no effect on the conditioned medium of M2a macrophages. *p* values were calculated using Student’s *t* test in GraphPad Prism 10.0 (GraphPad Software, San Diego, CA). (F) M2a conditioned medium promoted the growth of other cell lines: Calu-3 (lung cancer), MIA PaCa-2 (pancreatic cancer), HCT-116 (colon cancer), 22Rv1 (prostate cancer), and U-87MG (glioblastoma). Knockdown of PIP4K2A/2B in the M2a macrophage reduced these growth promoting activities (∗, *p* < 0.01; ∗∗, *p* < 0.001; ∗∗∗, *p* < 0.0001). Data are presented as mean ± SEM (*n* = 6). *p* values were calculated using Student’s *t* test in GraphPad Prism 10.0 (GraphPad Software, San Diego, CA).

We used the differentiated THP-1 cells as an *in vitro* model to study the role of PIP4K2A/2B in macrophage function.^47^^,^^48^ Within the complex tumor microenvironment, macrophages can dynamically adopt a range of activation states, with the classically activated M1 and alternatively activated M2 at the two phenotypic and functional extremes of a continuous spectrum.^49^^,^^50^^,^^51^ We generated a THP-1∗ cell line with tetracycline-inducible PIP4K2A/2B-targeting shRNA to circumvent the antiproliferative effect associated with PIP4K2A knockdown in undifferentiated parent THP-1 cells.^27^ After differentiation, the M0 macrophages were polarized into M1, M2a, M2b, M2c, and M2d states (Figure 6A).^52^^,^^53^ The supernatants of the macrophage cultures, especially those from M2b, stimulated the growth of H1975 cells (Figure 6B). DOX-induced knockdown of PIP4K2A/2B most drastically reduced the ability of M2a-conditioned medium to promote tumor cell growth (Figures 6C, 6D, and S3). Treating cultured M2a macrophages with 066ATZ, or another PIP4K2A inhibitor BAY-091,^36^ which shares the same ATP-competitive mechanism but has a different pharmacophore, eliminated the growth-promoting activity of the conditioned media (Figure 6E). Expressing a refractory PIP4K2A mutant in the M2a macrophage restored the growth promoting activity (Figure S4). To further rule out the possibility that off-target inhibition of CK2α/α′ by 066ATZ could contribute to this effect, M2a macrophages were also treated with CX-4945. At 0.5 μM, CX-4945 produced a similar degree of CK2α/α′ inhibition as 20 μM 066ATZ but had no effect on M2a macrophage’s growth promoting activity (Figure 6E). Taken together, these results are consistent with the idea that inactivation of PIP4K2A/2B alters the function of TAMs, thereby reducing their capacity to support tumor cell proliferation. The effect of PIP4K2A/2B inactivation on M2a-conditioned medium is not limited to H1975 cells. We tested five other tumor cell lines, harboring different oncogenic or tumor suppressor mutations and representing lung adenocarcinoma (Calu-3), undifferentiated pancreatic carcinoma (MIA PaCa-2), colon carcinoma (HCT-116), prostate carcinoma (22Rv1), and glioblastoma (U-87MG) and found them to respond similarly to the altered M2a-conditioned medium (Figure 6F).

## Discussion

The cellular functions of the PIP4Ks remain incompletely understood. Several recent studies have implicated PIP4K2A, which is exclusively cytosolic and catalytically most active, in the modification of membranes involved with the endo-lysosomal pathway and in lipid metabolism. Knockdown of PIP4K2A in fibroblasts disrupts membrane contacts between lysosome and peroxisome, interfering with cholesterol transport out of the lysosomes.^12^ In fasting animals, ablation of both PIP4K2A and PIP4K2B in hepatic cells blocks autophagosome-lysosome fusion, causing accumulation of lipid droplets and autophagic vesicles.^11^ Peroxisome dysfunction and impaired lipid trafficking negatively impact mitochondria structure and function.^19^ The resulting energy stress is made worse by glucose starvation, or by the inactivation of p53, a master transcription factor that, besides its well-known functions in controlling cell cycle, senescence, and apoptosis, also regulates cell energy homeostasis.^11^^,^^19^^,^^25^^,^^54^ These mechanistic delineations provided a conceptual foundation for targeting PIP4K in cancer: as has been demonstrated for genetically manipulated MEFs and multiple sarcoma and breast cancer cell lines, the metabolic vulnerability induced by PIP4K inactivation could be exploited to reduce cell survival and proliferation.^4^^,^^11^^,^^19^ Nevertheless, stratification of cancer types based on their sensitivity to PIP4K inhibition is still in its infancy, and the predictive value of p53 loss-of-function mutation is not yet adequately addressed. In contrast to the observation that knockdown of PIP4K2A/2B in sarcoma cells induces tumor regression,^19^ we and others found that PIP4K2A/2B inactivation had little impact on LUAD cells,^55^ regardless of their p53 status (H1975^mut^, H23^mut^, A549^wt^, and A427^wt^; Figure 4C). Neither did DOX-inducible knockdown of PIP4K2A/2B in H1975 cells alter the growth rate of xenograft tumor *in vivo* (Figure 4B). At the present time, it is difficult to reconcile these data with the finding that PIP4K2A^−/−^PIP4K2B^+/−^TP53^−/−^ mice, even at old age, never developed LUAD.^4^

An alternative explanation for the protective effect against LUAD, and possibly other cancers, upon whole-body PIP4K2A/2B inactivation could involve non-cell-autonomous mechanisms. Many components of the TME, including immune cells, fibroblasts, vasculature, various secreted factors, and extracellular matrix, profoundly influence tumor growth.^56^ The proliferation and immunosuppressive functions of Tregs, which are major pro-tumor immune cells within the TME, are already known to depend on PIP4K2B (and PIP4K2C).^28^ Although the nude mice used in our xenograft experiment lack T cells (thus Tregs), B cells, and NK cells, other immune cell types e.g., macrophages, dendritic cells, and neutrophils are still present and can potentially interact with the tumor. Indeed, IHC staining of harvested tumor samples revealed dense populations of macrophages at the periphery, and sometimes infiltrating deep into the center, of the xenograft tumors, thus implicating these immune cells as possible mediators of PIP4K inhibitor’s antitumor activity (Figure 5A). TAMs are highly heterogeneous and can influence tumor progression both positively and negatively.^45^^,^^46^ In the present study, we focused on soluble factors secreted by macrophages that can stimulate, or inhibit, the growth of tumor cells (Figure 6F). Of particular interest is the finding that PIP4K inactivation, by either genetic or pharmacological means, selectively eliminated the growth promoting activity of M2a macrophages (Figures 6C and 6E). The M2a, or alternatively activated, macrophages are induced by T helper 2 (T_H_2) cytokines IL-4 and IL-13 and usually involved in tissue repair, wound healing and immune regulation.^57^ Within TME, IL-4 can originate from tumor cells, T_H_2 cells, mast cells, and basophils, and we speculate that, in our experiment, the latter two cell types are the main source of this macrophage-polarizing cytokine. The co-localization of F4/80, a pan-macrophage marker, and CD206, which is abundantly expressed by M2a macrophages, supports the notion that, at least toward the end of the xenograft experiment, most macrophages have acquired this polarization state (Figure 5B). Future studies should aim to (1) validate the contribution of TAMs to the antitumor effects of PIP4K inhibition, (2) identify the macrophage-derived factor(s) responsible for the changes in growth-promoting activity observed in conditioned media (Figure S5), (3) elucidate the molecular pathways linking PIP4K activity to the function of macrophages derived from primary human monocytes, and (4) determine the relative roles of the two lipid kinase isoforms. Importantly, macrophages differentiated from THP-1 cells, a cancer-derived cell line, may not fully recapitulate the functional characteristics of tumor-associated macrophages, highlighting the value of studies using primary human monocyte–derived macrophages.

The slightly increased angiogenesis in the tumor upon PIP4K inhibitor treatment was unexpected (Figure 5C). At this time, we do not know if this is due to the inhibitor’s direct effect on endothelial cells or through a non-autonomous mechanism mediated by molecules secreted by other types of cells. Macrophages are known to produce factors that regulate blood vessel formation.^45^ Since enhanced circulation is expected to promote tumor growth, one may argue that PIP4K inhibitor’s antitumor activity, mediated through macrophages, could actually be stronger, if such an angiogenic side effect were not present. Combining PIP4K inhibitor with an anti-angiogenic agent therefore offers an appealing opportunity to enhance treatment efficacy.^58^

### Limitations of the study

Several mechanistic limitations of this study should be acknowledged. First, although our *in vivo* data support a non-tumor cell-autonomous mechanism for the tumor suppressive activity of the PIP4K inhibitor 066ATZ, we do not directly demonstrate that macrophages are strictly required for this effect *in vivo*, and contributions from other components of the TME cannot be excluded. Second, while we addressed potential off-target effects through rescue experiments using a drug-refractory PIP4K2A mutant and by demonstrating that the potent CK2 inhibitor CX-4945 lacks anti-tumor activity in the same xenograft model, additional targets engaged by small-molecule inhibitors in complex biological settings cannot be fully ruled out. Third, the compound’s *in vivo* and *in vitro* efficacy was evaluated using a single NSCLC cell line, and thus the generalizability of these findings to other NSCLC cell lines, tumor types, or genetic contexts remains to be determined. Finally, although multiplex cytokine and chemokine profiling was performed, PIP4K inhibition induced only modest changes in a limited subset of secreted factors, and the mechanistic links between these alterations and tumor growth suppression *in vivo* remain incompletely defined.

## Resource availability

### Lead contact

Requests for further information and resources should be directed to and will be fulfilled by the lead contact, Ya Ha (ya.ha@yale.edu).

### Materials availability

All unique/stable reagents generated in this study are available from the lead contact with a completed materials transfer agreement.

### Data and code availability

• Structural coordinates and X-ray diffraction data have been deposited at Protein DataBbank (accession code: 9OLE) and are publicly available as of the date of publication.
• This paper does not report original code.
• Any additional information required to reanalyze the data reported in this paper is available from the lead contact upon request.

## Acknowledgments

We thank Carla Rothlin and Sourav Ghosh for insights on tumor-associated macrophages. We thank David Stern, Samuel Katz, and Mark Lemmon for helpful suggestions. We thank Man Li, Jayalakshmi Lakshmipathi, and Yuping Qian for their technical assistance in the animal experiment. We thank the staff at APS beamline 24-ID C and NSLS-II beamline AMX/NYX for their assistance during X-ray diffraction data collection at the synchrotrons. We thank James Murphy and Brianna Duncan-Lowely for helping with onsite X-ray diffraction data collection at NSLS-II AMX/NYX. We thank Gary Rudnick for sharing his imager and oscillation counter, Ben Turk for sharing his radioactivity room, and Karen Anderson for sharing her plate reader and phosphorimager. We thank Tommy Cheng, Ben Turk, and Qin Yan for sharing cancer cell lines. This work was partly supported by 10.13039/100000002NIH grants GM138722 (to Y.H.), GM150502 (to Y.H.), and GM122473 (to J.E.); a YCMD pilot grant (to Y.H.); PITCH grant (to Y.H. and J.E.); an Evotec Bridge grant and Blavatnik Pilot grant (to Y.H. and J.E.); and the 10.13039/100015170Yale SPORE in Lung Cancer P50 CA196530.

## Author contributions

Conceptualization, Z.H., S.C., F.M., J.E., and Y.H.; data curation, Z.H., S.C., and Y.H.; formal analysis, Z.H., S.C., and Y.H.; funding acquisition, F.M., J.E., and Y.H.; investigation, Z.H., S.C., and Y.H.; methodology, Z.H., S.C., M.B., V.M., Y.X., H.W., F.M., A.C., C.B., M.V.Z., J.E., and Y.H.; project administration, F.M., J.E., and Y.H.; supervision, Y.H.; visualization, Z.H., S.C., and Y.H.; writing – original draft, Z.H., S.C., V.M., Y.X., M.V.Z., and Y.H.; ,riting – review and editing: Y.H.

## Declaration of interests

Yale University has filed a provisional patent application covering the compounds described in this study.

## STAR★Methods

### Key resources table

**Table:** 

REAGENT or RESOURCE	SOURCE	IDENTIFIER
**Antibodies**		

F4/80 monoclonal antibody (BM8) raised in rat	eBioscience	Cat#14-4801-82
CD206 polyclonal antibody raised in rabbit	Abcam	Cat#ab64693
Goat anti-rat Alexa Fluor 488 secondary antibody	Thermo Fisher Scientific	Cat#A-11006
Goat anti-rabbit Alexa Fluor 594 secondary antibody	Thermo Fisher Scientific	Cat#A-11012
PIP4K2A monoclonal antibody raised in rabbit	Cell Signaling Technology	Cat#5527
PIP4K2B polyclonal antibody raised in rabbit	Cell Signaling Technology	Cat#9694
CD31 antibody (IHC)	Yale Pathology Tissue Services	–
Goat anti-mouse IRDye 680RD secondary antibody	LI-COR Biosciences	Cat#926-68070
Goat anti-rabbit IRDye 800CW secondary antibody	LI-COR Biosciences	Cat#926-32211

**Bacterial and virus strains**		

*E. coli*: BL21 (DE3)	Agilent	230132
Lentiviral packaging system (psPAX2, pVSV-G)	Addgene	psPAX2 (#12260); pVSV-G (#8454)

**Biological samples**		

Xenograft tumor tissues (H1975∗)	This study	–

**Chemicals, peptides, and recombinant proteins**		

Tris-HCl	bioWORLD	–
Sodium chloride (NaCl)	americanBio	–
Magnesium chloride (MgCl_2_)	Sigma-Aldrich	Cat#M8266
Adenosine 5′-triphosphate (ATP)	Sigma-Aldrich	Cat#A2383
Hydrochloric acid (HCl)	Sigma-Aldrich	Cat#320331
Methanol	Sigma-Aldrich	Cat#34860
Chloroform	Sigma-Aldrich	Cat#C2432
Potassium oxalate	Sigma-Aldrich	Cat#P5645
Acetic acid	Sigma-Aldrich	Cat#A6283
Acetone	Sigma-Aldrich	Cat#34850
Imidazole	Sigma-Aldrich	Cat#I5513
Lysozyme	AMERSCO	Cat#0663-10G
Lithium sulfate (Li_2_SO_4_)	Sigma-Aldrich	Cat#L6375
HEPES buffer	Hampton Research	Cat#HR2-677
PEG 3,350	Hampton Research	Cat#HR2-529
Polybrene	Sigma-Aldrich	Cat#TR-1003
Puromycin	Sigma-Aldrich	Cat#P8833
PI(5)P lipid	Avanti Polar Lipids	–
[γ-^32^P]-ATP	PerkinElmer	–
Bovine brain extract (Folch Fraction I)	Sigma-Aldrich	Cat#B1502
Matrigel	Corning	Cat#354234
2-hydroxypropyl-β-cyclodextrin	Sigma-Aldrich	Cat#H107
Doxycycline	Sigma-Aldrich	Cat#D9891
Protease inhibitor cocktail	Sigma-Aldrich	Cat#P8340
Phosphatase inhibitor cocktail	Roche	Cat#4906845001
Recombinant human IFN-γ	PeproTech	Cat#300-02
Recombinant human IL-4	PeproTech	Cat#200-04
Recombinant human IL-10	PeproTech	Cat#200-10
Lipopolysaccharide (LPS) from *E. coli* O111:B4	Sigma-Aldrich	Cat#L2630
Ovalbumin (OVA), Grade V	Sigma-Aldrich	Cat#A5503
Anti-OVA IgG antibody	Sigma-Aldrich	Cat#C6534
5′-N-Ethylcarboxamidoadenosine (NECA)	Sigma-Aldrich	Cat#E2387
RIPA buffer	This study	–
Triton X-100	Sigma-Aldrich	Cat#T8787
Fluoromount-G with DAPI	SouthernBiotech	Cat#0100-20
Non-fat dry milk	americanBio	Cat#AB10109-01000
Glycerol	Sigma-Aldrich	Cat#G5516
Dimethyl sulfoxide (DMSO)	Sigma-Aldrich	Cat#D2650
XhoI restriction enzyme	New England Biolabs	Cat#R0146
EcoRI restriction enzyme	New England Biolabs	Cat#R0101
PIP4K2A recombinant protein	This study	
PIP4K2B recombinant protein	This study	
2-mercaptoethanol	Sigma-Aldrich	Cat#M6250

**Critical commercial assays**		

BCA Protein Assay Kit	Thermo Scientific	Cat#23225
CellTiter-Glo Luminescent Cell Viability Assay	Promega	Cat#G7570

**Deposited data**		

Crystal structure of PIP4K2A in complex with 066ATZ	Protein DataBank	PDB: 9OLE

**Experimental models: Cell lines**		

H1975	Gift from Yung-Chi Cheng lab	–
THP-1	Gift from Viswanathan Muthusamy lab	–
HEK293T	ATCC	–
MIA PaCa-2	Gift from Viswanathan Muthusamy lab	–
HCT-116	Gift from Viswanathan Muthusamy lab	–
22Rv1	Gift from Viswanathan Muthusamy lab	–
U-87MG	Gift from Viswanathan Muthusamy lab	–
Calu-3	Gift from Viswanathan Muthusamy lab	–

**Experimental models: Organisms/strains**		

Rag2/IL2RG double knockout mice (R2G2)	Envigo	R2G2

**Oligonucleotides**		

97-mer shRNA oligonucleotide (DKD): 5′-TGCTGTTGACAGTGAGCGAAGGTTTGGA ATTGATGATCAATAGTGAAGCCACAGATGTA TTGATCATCAATTCCAAACCTCTGCCTACT GCCTCGGA-3′	Wang et al.^55^	–
miRE-Xho-fw primer: 5′-TGAACTCGAGAAGG TATATTGCTGTTGACAGTGAGCG-3′	Wang et al.^55^	–
miRE-EcoOligo-rev primer: 5′-TCTCGAATTCT AGCCCCTTGAAGTCCGAGGCAGTAGGC-3′	Wang et al.^55^	–
miRseq5 primer: 5′-TGTTTGAATGAGG CTTCAGTAC-3′	Wang et al.^55^	–

**Recombinant DNA**		

LT3GEPIR vector	Fellmann et al.^59^	#111177
psPAX2 packaging plasmid	Addgene	#12260
pVSV-G envelope plasmid	Addgene	#8454

**Software and algorithms**		

HKL2000	HKL Research	–
CCP4i	CCP4	–
Coot	CCP4	–
GraphPad Prism	GraphPad Software	Version 10
PyMOL	Schrödinger	Version 2.5

**Other**		

Amicon Ultra centrifugal filter	Millipore	Cat#UFCB10024
PVDF membrane (Immobilon-FL)	Millipore	Cat#IPFL00010
NuPAGE gel system	Invitrogen	Cat#NP0321BOX
Odyssey CLx imaging system	LI-COR Biosciences	–
Molecular Imager FX phosphorimager	Bio-Rad	–
TLC silica gel plates	Millipore	Cat#105553

### Experimental model and study participant details

Seven-week-old female Rag2/IL2RG double knockout (R2G2) mice were purchased from Envigo and housed under a 12-h light/12-h dark cycle in a temperature- and humidity-controlled facility maintained by the Yale Animal Resources Center (YARC), with *ad libitum* access to food and water.

Human cell lines H1975, Calu-3, MIA PaCa-2, HCT-116, 22Rv1, THP-1, U-87MG, and HEK293T cells were used in this study. The H1975 cell line was a gift from the laboratory of Dr. Yung-Chi Cheng, and Calu-3, MIA PaCa-2, HCT-116, 22Rv1, THP-1, U-87MG cell lines were a gift from the laboratory of Dr. Viswanathan Muthusamy. Cell lines were not further authenticated by the authors. All cell lines were routinely tested for mycoplasma contamination and confirmed to be negative. Cells were cultured at 37°C in a humidified incubator with 5% CO2. Tetracycline-inducible knockdown cell lines targeting PIP4K2A and PIP4K2B were generated using lentiviral transduction followed by antibiotic selection and monoclonal isolation with a detailed protocol described below.

### Method details

#### Chemical synthesis

066ATZ was synthesized in eight steps from commercially available (2R)-2-amino-3-(pyridin-2-yl)propanoic acid hydrochloride **1**. Treatment with thionyl chloride in methanol gave quantitative conversion to methyl ester **2**. Reductive amination with cyclopentanone and sodium triacetoxyborohydride gave amine **3** which was treated with 2,4-dichloro-5-nitropyrimidine to give chloropyrimidine **4**. Reduction of the nitro group with iron and acetic acid gave cyclized product **5**. Alkylation with methyl iodide and sodium hydride gave intermediate **6**. Treatment with trimethylsilylbrodide in propionitrile at 150 °C gave bromine substituted product **7**. Treatment with 5-aminothiazole-2-carbonitrile and cesium carbonate with zantphos and Pd_2_(dba)_3_ in dioxane at 100 °C gave thiazole **8**. Finally, formation of the tetrazole with sodium azide in DMF at 115 °C gave target compound **9** (066ATZ).

#### Synthetic scheme

**Figure:**
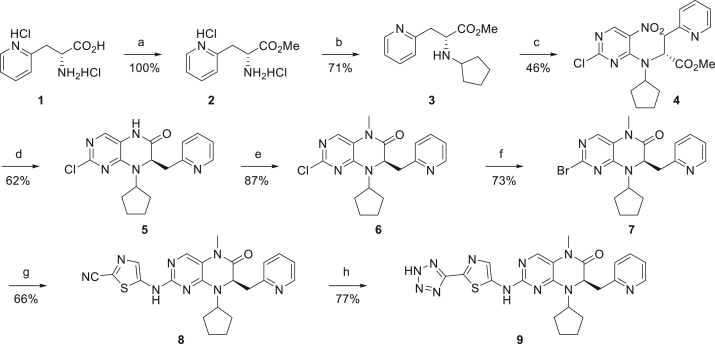

*Reagents and conditions.* (a) SOCl_2_, MeOH (0°C to reflux), 5 h; (b) CH_3_COONa, cyclopentanone, NaBH(OAc)_3_, CH_2_Cl_2_, 0°C–20°C; (c) 2,4-dichloro-5-nitropyrimidine, K_2_CO_3_, acetone 0°C–20°C; (d) Fe, AcOH, 75°C, 1 h; (e) CH_3_I, NaH, DMF -10°C - 20°C, 4 h; (f) TMSBr, propionitrile, 150°C, 30 h, (g) 5-aminothiazole-2-carbonitrile, Cs_2_CO_3_, xantphos, Pd_2_(dba)_3_, dioxane, 100°C, 18 h; (h) NaN_3_, NH_4_Cl, DMF, 115°C, 4 h.

#### Detailed experimental procedures

##### Methyl (R)-2-amino-3-(pyridin-2-yl)propanoate dihydrochloride (**2**)

**Figure:**
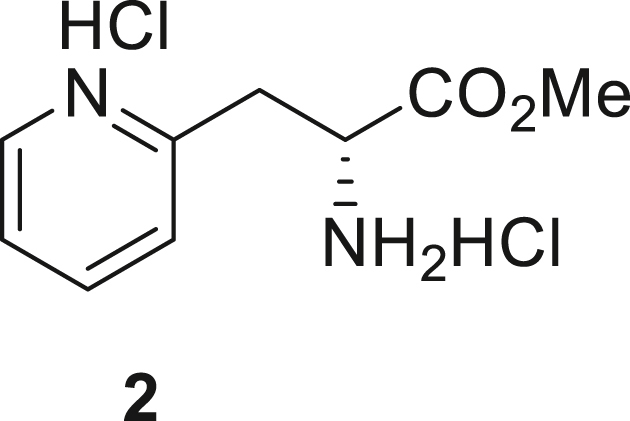

A stirred heterogeneous solution of (2R)-2-amino-3-(pyridin-2-yl)propanoic acid dihydrochloride, **1** (, 1 eq., 15 g, 62.74 mmol) in MeOH (150 mL) was cooled to 0 °C and treated with thionyl chloride (2.5 eq., 18.66 g, 11.38 mL, 156.84 mmol) dropwise over 15 min. After the addition was complete, the cooling bath was removed and the reaction mixture was heated to reflux for 4 h. After cooling to room temperature, the solution was concentrated under reduced pressure and stirred with ether (150 mL) for 30 min. The precipitated product was isolated by filtration and dried under high vacuum to give methyl (2R)-2-amino-3-(pyridin-2-yl)propanoate dihydrochloride **2** (16 g, 100%) as white solid, which was used without further purification. ^1^H NMR (400 MHz, D_2_O) δ 3.51–3.53 (m, 2H), 3.59 (s, 3H), 4.51 (t, J = 7.5 Hz, 1H), 7.77 (d, J = 7 Hz, 1H), 7.82 (d, J = 8 Hz, 1H), 8.33 (t, J = 8 Hz, 1H), 8.55 (d, J = 6 Hz, 1H).

##### Methyl (R)-2-(cyclopentylamino)-3-(pyridin-2-yl)propanoate (**3**)

**Figure:**
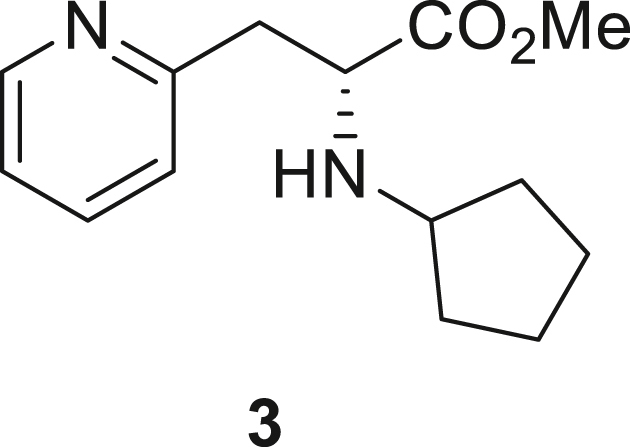

To a stirred solution of methyl (2R)-2-amino-3-(pyridin-2-yl)propanoate dihydrochloride **2** (1 eq., 16 g, 63.21 mmol) and cyclopentanone (2.1 eq., 11.17 g, 11.75 mL, 132.74 mmol) in dichloromethane (160 mL) was cooled to 0 °C (ice-bath) and treated with sodium acetate (2 eq., 10.37 g, 126.42 mmol) and sodium triacetoxyborohydride (1.5 eq., 20.095 g, 94.82 mmol). After stirring for 1 h, the cooling bath was removed and the reaction mixture was allowed to warm to room temperature with continued stirring overnight. The resulting heterogeneous solution was re-cooled in an ice-bath and quenched by careful addition of saturated aqueous sodium bicarbonate. The layers were separated and the aqueous layer was extracted with dichloromethane (3 x 50 mL). The organic layer was dried over sodium sulfate, filtered and concentrated. The residue was purified by silica gel flash column chromatography (eluent: 50% ethyl acetate in hexanes, ethyl acetate, 1–4% MeOH in ethyl acetate) to give methyl (2R)-2-(cyclopentylamino)-3-(pyridin-2-yl)propanoate **3** (11.15 g, 71%) as brown oil. ^1^H NMR (400 MHz, CDCl3) δ 1.24-2-04 (m, 8H), 3.09–3.32 (m, 3H), 3.73 (s, 3H), 3.81–3.85 (m, 1H), 7.38–7.02 (m, 3H), 7.59 (td, J = 7.7, 1.7 Hz, 1H), 8.51 (d, J = 4.9 Hz, 1H). Mass (m/z): calculated for: C_14_H_20_N_2_O_2_ 248, found 249 (M + H).

##### Methyl (R)-2-((2-chloro-5-nitropyrimidin-4-yl)(cyclopentyl)amino)-3-(pyridin-2-yl)propanoate (**4**)

**Figure:**
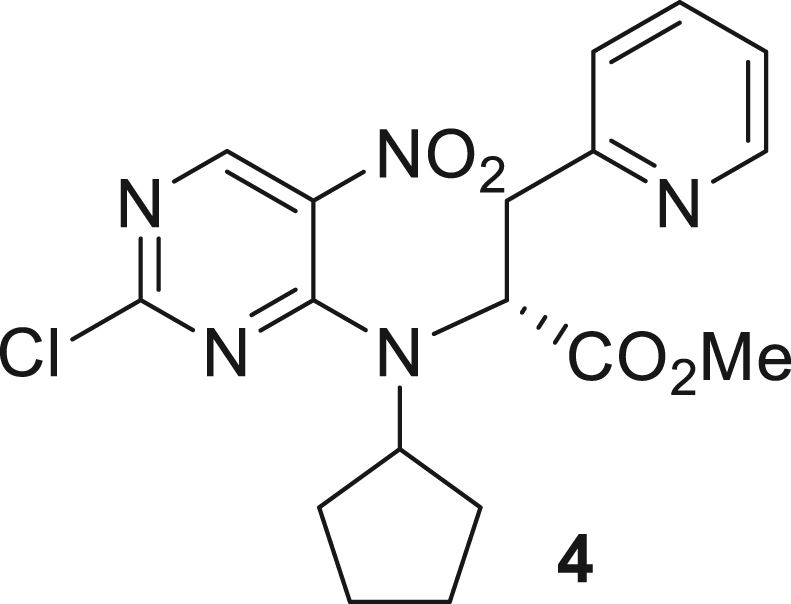

While under nitrogen, an ice-cold, stirred solution of methyl (2R)-2-(cyclopentylamino)-3-(pyridin-2-yl)propanoate **3** (1 eq., 11.1 g, 44.7 mmol) in acetone (110 mL) was treated with potassium carbonate (1.1 eq., 6.8 g, 49.17 mmol) and a solution of 2,4-dichloro-5-nitropyrimidine (1.1 eq., 9.54 g, 49.17 mmol) in acetone (90 mL). Once the addition was complete, the cooling bath was removed and the reaction mixture was stirred overnight. The reaction mixture was concentrated under reduced pressure, diluted with water (200 mL) and extracted with ethyl acetate (3 x 100 mL). The combined organic layer was dried over sodium sulfate, filtered and concentrated under reduced pressure. The crude product was purified by silica gel flash column chromatography (eluent: 0–25% ethyl acetate in hexanes) to give methyl (2R)-2-[(2-chloro-5-nitropyrimidin-4-yl)(cyclopentyl)amino]-3-(pyridin-2-yl)propanoate **4** (8.3 g, 46%) as light orange resin. ^1^H NMR (400 MHz, CDCl3) δ 1.51–1.77 (m, 8H), 3.40 (dd, J = 15.6, 6.3 Hz, 1H), 3.64 (s, 3H), 3.88 (dd, J = 15.6, 6.3 Hz, 1H), 3.75–3.96 (m, 1H), 4.74 (s, 1H), 7.21 (d, J = 8 Hz, 2H), 7.67 (t, J = 7.7 Hz, 1H), 8.52 (d, J = 4.7 Hz, 1H), 9.28 (s, 1H).

##### (R)-2-Chloro-8-cyclopentyl-7-(pyridin-2-ylmethyl)-7,8-dihydropteridin-6(5H)-one (**5**)

**Figure:**
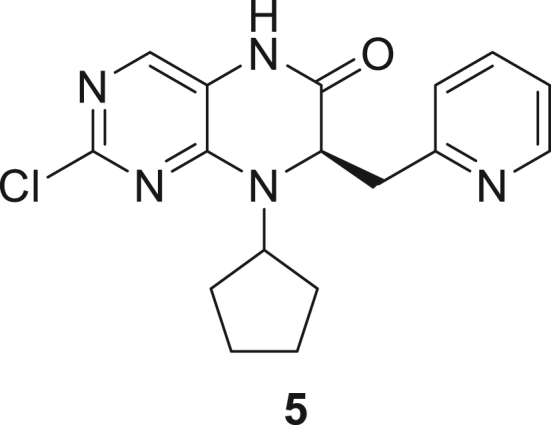

A stirred solution of methyl (2R)-2-[(2-chloro-5-nitropyrimidin-4-yl)(cyclopentyl)amino]-3-(pyridin-2-yl)propanoate **4** (1 eq., 10.5 g, 25.87 mmol) in acetic acid (105 mL) was heated to 75 °C and treated with iron (2.5 eq., 3.61 g, 0.46 mL, 64.68 mmol) portion-wise. After stirring for 1 h, the heating bath was removed and the reaction mixture was allowed to cool to room temperature. The resulting mixture was filtered through Celite, washing the Celite thoroughly with ethyl acetate and finally with MeOH. The filtrate was concentrated and purified by silica gel flash column chromatography (eluent: ethyl acetate, 0.5–2% MeOH in ethyl acetate) to give (7R)-2-chloro-8-cyclopentyl-7-[(pyridin-2-yl)methyl]-5,6,7,8-tetrahydropteridin-6-one **5** (5.5 g, 62%) as light brown solid. ^1^H NMR (400 MHz, CDCl3) δ 2.26–1.57 (m, 8H), 3.27 (dd, J = 13.9, 8.2 Hz, 1H), 3.50 (dd, J = 13.8, 4.3 Hz, 1H), 4.37–4.58 (m, 1H), 4.74 (dd, J = 8.2, 4.2 Hz, 1), 7.32 (d, J = 8 Hz, 1H), 7.37 (d, J = 7.2 Hz, 1H), 7.54 (s, 1H), 7.81 (t, J = 7.7 Hz, 1H), 8.27 (s, 1H), 8.49 (d, J = 5.2 Hz, 1H).

##### (R)-2-Chloro-8-cyclopentyl-5-methyl-7-(pyridin-2-ylmethyl)-7,8-dihydropteridin-6(5H)-one (**6**)

**Figure:**
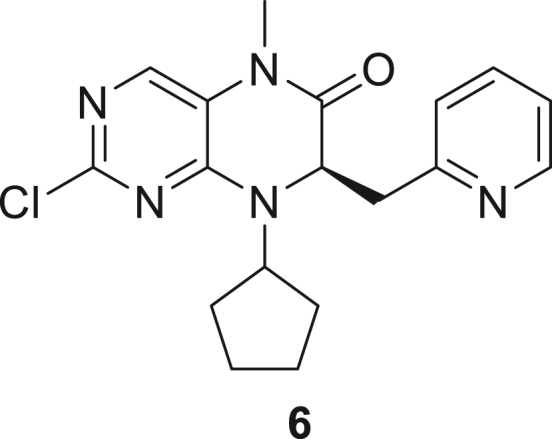

A stirred solution of (7R)-2-chloro-8-cyclopentyl-7-[(pyridin-2-yl)methyl]-5,6,7,8-tetrahydropteridin-6-one **5** (1 eq., 5.5 g, 16 mmol) and iodomethane (1.3 eq., 2.95 g, 1.29 mL, 20.8 mmol) in DMF (55 mL) was cooled to −10 °C and treated with sodium hydride (1.3 eq., 0.83 g, 60% in mineral oil, 20.8 mmol) portion-wise. After stirring for 30 min, the cooling bath was removed and reaction mixture was allowed to warm to room temperature. After stirring an additional 3 h at RT, the reaction mixture was cooled in an ice-bath and quenched by the careful addition of cold water (200 mL). The resulting mixture was extracted with ethyl acetate (5 x 50 mL). The combined organic layer was dried over sodium sulfate and concentrated. The crude product was purified by silica gel flash column chromatography (eluent: ethyl acetate, 1–2% MeOH in ethyl acetate) to give (7R)-2-chloro-8-cyclopentyl-5-methyl-7-[(pyridin-2-yl)methyl]-5,6,7,8-tetrahydropteridin-6-one **6** (5 g, 13.97 mmol, 87%) as cream colored solid. 1H NMR (400 MHz, CDCl3) δ 1.62–2.09 (m, 8H), 3.11 (dd, J = 13.9, 8 Hz, 1H), 3.19 (s, 3H), 3.38 (dd, J = 13.9, 4 Hz, 1H), 4.41–4.47 (m, 1H), 4.75 (dd, J = 6.5, 4 Hz, 1H), 6.95 (d, J = 8 Hz, 1H), 7.02 (dd, J = 8, 4 Hz, 1H), 7.31 (s, 1H), 7.43 (t, J = 8 Hz, 1H), 8.39 (d, J = 5 Hz, 1H). Mass (m/z) calculated for C_18_H_20_ClN_5_O 357, found 358 (M + H).

##### (R)-2-Bromo-8-cyclopentyl-5-methyl-7-(pyridin-2-ylmethyl)-7,8-dihydropteridin-6(5H)-one (**7**)

**Figure:**
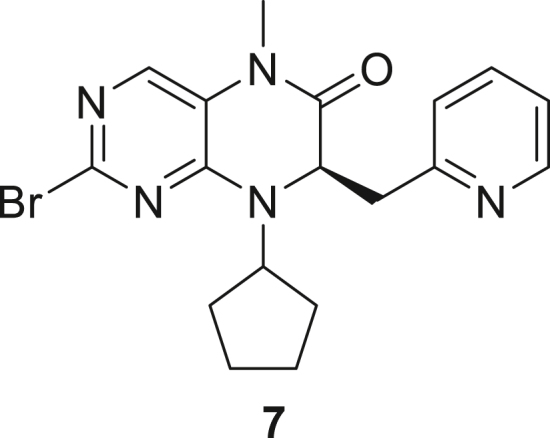

A stirred solution of (7R)-2-chloro-8-cyclopentyl-5-methyl-7-[(pyridin-2-yl)methyl]-5,6,7,8-tetrahydropteridin-6-one **6** (1 eq., 5 g, 13.97 mmol) in propionitrile (55 mL) in a pressure flask, was treated with bromotrimethylsilane (5 eq., 10.7 g, 9.22 mL, 69.86 mmol) and the flask was capped. It was placed in an oil bath at 150 °C. After stirring for 4 h the solution was cooled to room temperature, opened and treated with additional bromotrimethylsilane (1.085 eq., 2.32 g, 2 mL, 15.15 mmol) and re-heated to 150 °C for 4 h. The reaction mixture was stirred overnight at room temperature, opened and treated with additional bromotrimethylsilane (1.085 eq., 2.32 g, 2 mL, 15.15 mmol). The solution was re-heated to 150 °C for 4 h. After cooling to room temperature the solution was concentrated, dissolved in saturated aqueous sodium bicarbonate (100 mL) and extracted with ethyl acetate (3 x 50 mL) and then with dichloromethane (3 x 25 mL). The combined organic layer was dried over sodium sulfate and concentrated. The residue was purified by silica gel flash column chromatography (eluent:0–5% MeOH in ethyl acetate) to give (7R)-2-bromo-8-cyclopentyl-5-methyl-7-[(pyridin-2-yl)methyl]-5,6,7,8-tetrahydropteridin-6-one **7** (4.1 g, 73%) as off-white solid. ^1^H NMR (400 MHz, CDCl3) δ 1.58–2.23 (m, 8H), 3.15 (dd, J = 13.8, 4 Hz, 1H), 3.16 (s, 3H), 3.33 (dd, J = 13.8, 4 Hz, 1H), 4.36–4.48 (m, 1H), 7.72 (dd, J = 5.8, 4.0 Hz, 1H), 6.98 (d, J = 8 Hz, 1H), 7.05 (td, J = 8, 4 Hz, 1H), 7.29 (s, 1H), 7.46 (td, J = 7.7, 2.2 Hz, 1H), 8.35 (d, J = 4.8 Hz, 1H). Mass (m/z) calculated for C_18_H_20_BrN_5_O 401, found 402 (M + H).

##### (R)-5-((8-Cyclopentyl-5-methyl-6-oxo-7-(pyridin-2-ylmethyl)-5,6,7,8-tetrahydropteridin-2-yl)amino)thiazole-2-carbonitrile (**8**)

**Figure:**
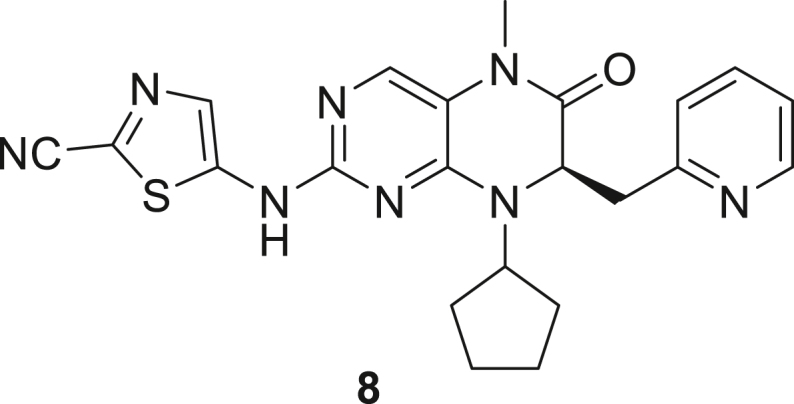

While under nitrogen, a solution of (7R)-2-bromo-8-cyclopentyl-5-methyl-7-[(pyridin-2-yl)methyl]-5,6,7,8-tetrahydropteridin-6-one **7** (1 eq., 4.1 g, 10.19 mmol) and 2-amino-1,3-thiazole-5-carbonitrile (1.8 eq., 2.3 g, 18.34 mmol) in dioxane (41 mL) was degassed with nitrogen and treated with cesium carbonate (3 eq., 9.96 g, 30.57 mmol), xantphos (0.3 eq., 1.77 g, 3.057 mmol) and tris (dibenzylideneacetone) dipalladium (0) (0.1 eq., 0.93 g, 1.019 mmol). The reaction vessel was capped and heated at 100 °C for 18 h. The reaction mixture was cooled to room temperature and the solvent was removed under reduced pressure. Water was added to the residue and this aqueous phase was sonicated with ethyl acetate (100 mL) and the organic layer was separated. The aqueous layer was extracted 2 more times with ethyl acetate (100 mL each). The insoluble material present was separated by filtration and sonicated with dichloromethane (3 x 50 mL). The dichloromethane layer was mixed with ethyl acetate extracts. The aqueous layer was then extracted dichloromethane (3 x 50 mL). The combined organic layer was then dried over sodium sulfate and concentrated. The residue was then triturated with 1:1 mixture of ethyl acetate and MeOH (50 mL). The solid was isolated by filtration to give 2-[[(7R)-8-cyclopentyl-5-methyl-6-oxo-7-[(pyridin-2-yl)methyl]-5,6,7,8-tetrahydropteridin-2-yl]amino]-1,3-thiazole-5-carbonitrile **8** (3 g, 6.72 mmol, 66%) as beige solid. ^1^H-NMR (400 MHz, CDCl3): ẟ 1.68–2.27 (m, 8H), 3.14 (dd, J = 13.7, 6.3 Hz, 1H), 3.25 (s, 3H), 3.31 (dd, J = 13.6, 4.3 Hz, 1H), 4.62–4.81 (m, 2H), 6.99 (d, J = 7.8 Hz, 1H), 7.06 (dd, J = 7.6, 4.9 Hz, 1H), 7.46 (td, J = 7.7, 1.8 Hz, 1H), 7.64 (s, 1H), 7.99 (s, 1H), 8.37 (d, J = 4.9 Hz, 1H). Mass (m/z) calculated for C_22_H_22_N_8_OS 446, found 447 (M + H).

##### (R)-2-((2-(2H-tetrazol-5-yl)thiazol-5-yl)amino)-8-cyclopentyl-5-methyl-7-(pyridin-2-ylmethyl)-7,8-dihydropteridin-6(5H)-one (**9** or 066ATZ)

**Figure:**
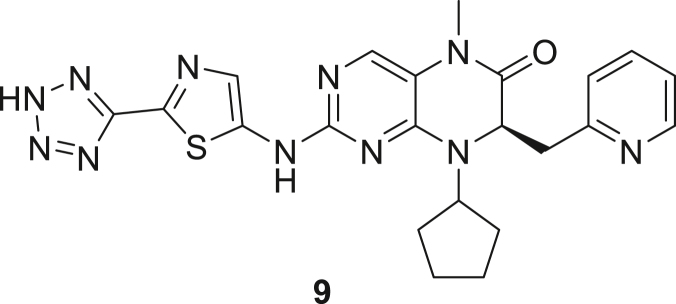

Side by side, two heterogeneous mixtures of 2-[[(7R)-8-cyclopentyl-5-methyl-6-oxo-7-[(pyridin-2-yl)methyl]-5,6,7,8-tetrahydropteridin-2-yl]amino]-1,3-thiazole-5-carbonitrile **8** (1 eq., 1.3 g, 2.91 mmol), sodium azide (10 eq., 1.89 g, 1.023 mL, 29.11 mmol), and ammonium chloride (10 eq., 1.56 g, 1.025 mL, 29.11 mmol) in DMF (13 mL) were heated to 115 °C in a pressure tube for 4h. After cooling to room temperature, the reaction mixtures were combined and the solids were removed by filtration. The filtrate was concentrated under reduced pressure. The residue was triturated with a mixture of 20 mL of ether and 10 mL of ethyl acetate. The solid was isolated by filtration. This solid was then dissolved ethyl acetate (20 mL) and MeOH (20 mL). The insoluble material was removed by filtration. The filtrate was concentrated under reduced pressure. The residue was filtered through a small silica gel column (eleuent:5%, 10% MeOH in dichloromethane containing a drop of AcOH, then 25%, 50% EtOH in dichloromethane with a drop of AcOH) to give 2.2 g of (7R)-8-cyclopentyl-5-methyl-7-[(pyridin-2-yl)methyl]-2-[[5-(2H-1,2,3,4-tetrazol-5-yl)-1,3-thiazol-2-yl]amino]-5,6,7,8-tetrahydropteridin-6-one **9** (066ATZ) (2.28 g, 77%) as off-white solid. ^1^H-NMR (400 MHz, DMSO-d6): ẟ 1.68–1.93 (m, 8H), 3.05–3.19 (m, 2H), 3.15 (s, 3H), 4.58–4.72 (m, 1H), 4.73 (dd, J = 6.2, 4.2 Hz, 1H), 7.08 (d, J = 8 Hz, 1H), 7.12 (dd, J = 8, 4 Hz, 1H), 7.54 (t, J = 8 Hz, 1H), 7.69 (s, 1H), 7.95 (s, 1H), 8.33 (d, J = 4.9 Hz, 1H), 11.48 (brs, 1H). Mass (m/z) calculated for C_22_H_23_N_11_OS 489, found 490 (M + H).

#### Crystallography

Recombinant PIP4K2A (residues 33–405) was crystallized in 0.2 M Li_2_SO_4_, 0.1 M HEPES pH 7.5, 25% PEG3,350 (the index G4 condition) as previously described.^25^ The apo crystals were soaked with 0.5 mM 066ATZ overnight to generate the inhibitor complex. 20% glycerol was used for cryoprotection. X-ray diffraction data were collected at APS beamline 24-ID E, NSLS-II beamlines AMX and NYX and processed by *HKL2000* (Table S1).^60^ The apo structure (PDB entry 7N6Z) was used as the initial model for rigid body refinement and difference Fourier map calculation by *CCP4i*.^25^^,^^61^ A starting model of 066ATZ was generated by *cprodrg*.^62^ *Coot* and *CCP4i* were used for modeling and refinement.^63^ All structural figures in the paper were generated by *PyMOL* (Schrödinger, LLC. *The PyMOL Molecular Graphics System*, Version 2.5).

#### Tetracycline-inducible knockdown cell lines

The tetracycline-inducible shRNA knockdown system was constructed from LT3GEPIR (Addgene, plasmid #111177), which contains an optimized tetracycline-on miR-E backbone for inducible expression of shRNA.^59^ The lentiviral vector includes sequences for a GFP reporter, a control shRNA targeting renilla luciferase (Ren), and the puromycin resistance marker. The sequence targeting both PIP4K2A and PIP4K2B (DKD)^55^ was PCR-amplified using primers miRE-Xho-fw and miRE-EcoOligo-rev from the 97-mer oligonucleotide template (key resources table), and sub-cloned into LT3GEPIR via double digestion with XhoI/EcoRI and ligation by T4 DNA ligase (New England Biolabs). Primer miRseq5 was used to confirm the sequence of the lentiviral vector. Lentiviral particles were generated by transfecting 11 μg of the target plasmid, along with 9 μg of the psPAX2 packaging plasmid and 7 μg of the pVSV-G envelope plasmid, into HEK293T cells cultured in a 100 mm^2^ dish. After 18 h of incubation, the medium was replaced with fresh culture medium. Supernatant was collected 48 h post-transfection, centrifuged at 1,000 × g for 5 min, and filtered through a 0.45 μm filter to obtain the first batch of lentivirus. The viral supernatant was subsequently concentrated using a 100,000 MWCO centrifugal filter unit (Amicon, UFCB10024) at 4,700 rpm to a final volume of ∼500 μL and aliquoted into 30 μL portions. For transduction, each 30 μL aliquot was diluted 1:150 in RPMI-1640 medium (Gibco, 11875-093) supplemented with 10% fetal bovine serum (Gibco, 26140079) and 10 μg/mL polybrene. A total of 50,000 H1975 cells were seeded into each well of a 6-well plate containing the diluted virus. After 48 h, cells were selected with 1 μg/mL puromycin to enrich for transduced populations (H1975-DKD). For monoclonal selection (H1975∗), H1975-DKD cells were diluted to 500 cells/mL and 100 μL per well was plated into 96-well plates with puromycin-containing medium. THP-1∗ cells with tetracycline-inducible knockdown of *PIP4K2A* and *PIP4K2B* were generated using a similar approach. Because THP-1 cells grow in suspension, transfected cells were enriched by repeated subculturing in puromycin-containing medium, allowing selection for puromycin-resistant cells until a predominantly viable population was obtained.

#### Kinase assay

The kinase activities of PIP4K2A and PIP4K2B in the presence of the inhibitor were assessed by measuring the production of radiolabeled PI(4,5)P_2_ as previously described.^64^ The inhibitor was prepared as 10 mM stock solutions in DMSO and stored at −80°C. Reactions were performed in a 50 μL assay mixture containing 10 mCi [γ-^32^P]-ATP, 40 μM PI(5)P, 0.4 μg/mL PIP4K2A, 20 μM ATP, 10 mM MgCl_2_, and 20 mM HEPES (pH 7.3), and incubated at room temperature for 1 h. For the less active PIP4K2B, reactions were carried out using 4 μg/mL enzyme for 3 h at room temperature. Prior to initiating the kinase reaction, inhibitor was pre-incubated with the respective enzymes for 20 min at room temperature. The reactions were quenched by the addition of 5 μL of 12 N HCl, followed by the addition of 175 μL PBS and 380 μL of a 1:1 (v/v) methanol/chloroform mixture containing 10 μg/mL bovine brain extract (Type I, Folch Fraction I; Sigma-Aldrich) for lipid extraction. The lower organic phase containing lipids was collected and dried under vacuum. Dried lipids were resuspended in 25 μL of methanol/chloroform (2:1, v/v) and separated via thin-layer chromatography (TLC) on heat-activated, 1% potassium oxalate-coated silica gel 60 plates (20 × 20 cm, Merck). TLC plates were developed using a solvent system consisting of water, acetic acid, methanol, acetone, and chloroform in a 3:32:24:30:64 ratio (v/v). Radiolabeled PI(4,5)P_2_ products were visualized and quantified using a phosphorimager (Bio-Rad Molecular Imager FX).

#### Immunoblotting

Cells were washed with ice-cold 1× DPBS and lysed in RIPA buffer containing 50 mM Tris-HCl (pH 8.0), 150 mM NaCl, 1 mM EGTA, 1% NP-40, 0.5% sodium deoxycholate, 0.1% SDS, and supplemented with protease inhibitors (Sigma-Aldrich) and phosphatase inhibitor cocktails (Roche). Total protein concentration was quantified using the BCA Protein Assay Kit (Thermo Scientific). Equal amounts of protein were separated by SDS-PAGE (NuPAGE, Invitrogen) and transferred onto 0.45 μm PVDF membranes (Immobilon-FL, Millipore). Membranes were blocked with 5% non-fat milk (americanBIO) and incubated overnight at 4°C with the appropriate primary antibodies. After washing, membranes were incubated with fluorescently labeled secondary antibodies (LI-COR Biosciences) for 1 h at room temperature. Protein signals were visualized using the Odyssey CLx imaging system (LI-COR Biosciences).

#### Animal studies

All animal procedures were performed in compliance with institutional and national regulations and were approved by the Institutional Animal Care and Use Committee (IACUC) of Yale University (protocol #: 2024–20218). H1975∗ cells were cultured in RPMI-1640 medium (Gibco, 11875-093) supplemented with 10% fetal bovine serum (Gibco, 26140079) and 1 μg/mL puromycin. Cells were harvested, resuspended in culture medium containing 50% Matrigel (Corning), and 1 × 10^7^ cells were injected subcutaneously into each mouse to establish xenograft tumors. Mice were randomly assigned to treatment groups based on tumor growth. Tumor volumes were measured every three days following implantation. Mice were euthanized by isoflurane overdose followed by organ collection when tumor volume reached 1,000 mm^3^. Tumor tissues were harvested and either formalin-fixed or snap-frozen in a dry ice/ethanol bath. For protein analysis, 50–80 mg of tumor tissue was homogenized in 500–800 μL RIPA buffer supplemented with protease (Sigma-Aldrich) and phosphatase (Roche) inhibitor cocktails. Lysates were clarified by centrifugation at 12,000 rpm for 10 min and stored at −80°C. For studies involving PIP4K2A/2B knockdown, mice were administered either regular drinking water or water containing 1 mg/mL doxycycline starting on the day of tumor implantation; water was replaced every six days. In studies evaluating 066ATZ, 20% 2-hydroxypropyl-β-cyclodextrin was used as the vehicle. 066ATZ was dissolved in vehicle at 25 mg/mL and administered intraperitoneally (IP) at 100 mg/kg, five days per week.

#### Immunofluorescence

The FFPE slides were deparaffinized, subjected to heat-mediated antigen retrieval, rehydrated (similar as IHC and H&E staining), and permeabilized using PBST containing 0.1% Triton X-100 for 30 min. Slides were then incubated with protein blocking buffer for an additional 30 min. Primary antibodies were diluted in dilution buffer and incubated overnight at 4°C. The primary antibodies used were rat anti-F4/80 (BM8, eBioscience, 5 μg/mL) and rabbit anti-CD206 (ab64693, Abcam, 1 μg/mL). After washing three times with PBST, tissues were incubated with corresponding secondary antibodies for 1 h at room temperature. The secondary antibodies used included goat anti-rat Alexa Fluor 488 (A-11006, Thermo Fisher, 2 μg/mL) and goat anti-rabbit Alexa Fluor 594 (A-11012, Thermo Fisher, 2 μg/mL). Following another series of PBST washes, slides were mounted using mounting solution containing DAPI. Imaging was performed using a KEYENCE BZ-X Fluorescence Microscope.

### Quantification and statistical analysis

Statistical analyses were conducted using Prism 10 software (GraphPad). The specific statistical tests applied, sample sizes, and the notation of asterisks are detailed in the corresponding figure legends. *p* values were calculated using Student’s *t* test in GraphPad Prism 10.0 (GraphPad Software, San Diego, CA).
