# Supplementary material for: PIP4K2A/2B inhibitor suppresses tumor growth in a xenograft model of NSCLC
Source: iScience. 2026 Apr 30;29(6):115952. doi: 10.1016/j.isci.2026.115952 (PMC13206749; doi:10.1016/j.isci.2026.115952)
Supplement: Document S1. Figures S1–S5 and Table S1 [file mmc1.pdf]

iScience, Volume *29*

Supplemental information

PIP4K2A/2B inhibitor suppresses tumor growth in a xenograft model of NSCLC

Zunyu He, Song Chen, Marcus Bosenberg, Viswanathan Muthusamy, Yibo Xi, He Wang, Fabrizio Micheli, Agostino Cianciulli, Claudia Beato, Michael Van Zandt, Jonathan Ellman, and Ya Ha

Table S1. Crystallographic statistics for PIP4K2A in complex with 066ATZ (space group: $P6_122$).

Data Collection	
Wavelength (Å)	0.979
Cell Dimensions (Å)	a=b=136.5, c=94.9
[a]Resolution (Å)	40-2.4 (2.49-2.40)
Redundancy	10.8
Completeness (%)	99.9
<I/σ>	12.8
[a,b]R_{merge}	0.064 (0.959)
Refinement	
Unique reflections	20,802
Number of Atoms	
Protein	2,490
Ligand	35
Solvent	212
[c]R_{work}/R_{free}	0.184/0.243
B-factor ($Å^2$)	79
r.m.s. deviations	
Bond lengths (Å)	0.005
Bond angles (°)	1.281
PDB accession code	9OLE

[a]Highest resolution shell is shown in parentheses.

[b]$R_{merge} = \sum | I_i - \langle I \rangle | / \sum I_i$

[c]$R_{work} = \sum | F_o - F_c | / \sum F_o$. R_{free} is the cross-validation R factor for the test set of reflections (5% of the total) omitted in model refinement.

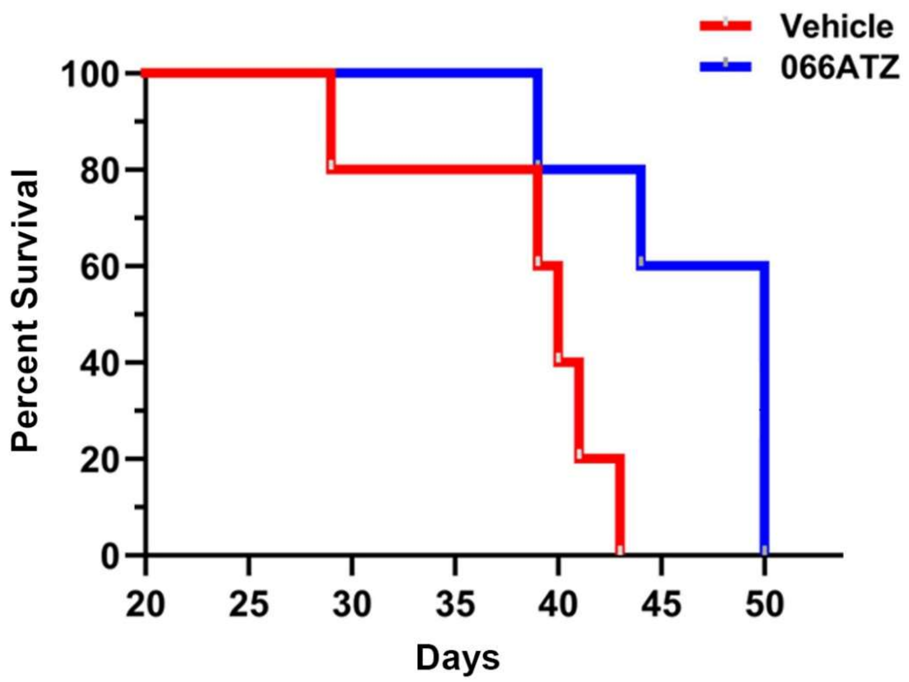

Figure S1. Kaplan-Meier survival curve of tumor-bearing R2G2 mice treated with either vehicle or 100 mg/kg 066ATZ (n=5; Fig. 4A). The death of one animal in the treatment group on day 39 is unrelated to the xenograft tumor (tumor size ~550 mm^3). 066ATZ significantly improved survival based on the Mantel-Cox log-rank test ($p=0.02$).

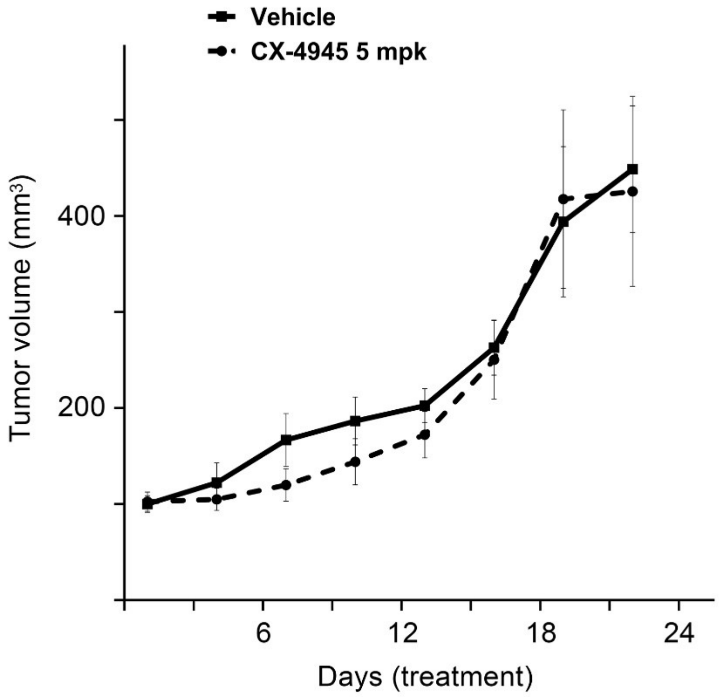

Figure S2. CK2α/α' inhibitor CX-4945 does not affect H1975* xenograft tumor growth rate. The growth curves represent the average of five animals in the treatment or control arm. Data are presented as mean ± SEM (n=5). CX-4945 (5 mg/kg) or vehicle was IP injected 5 times a week after the tumor reached ~100 mm^3. Since there is no obvious difference in tumor size, all mice were sacrificed 22 days after initiating the IP injections. CX-4945 has a K_d ~0.4 nM against CK2α or CK2α',[1,2] thus about 100-fold more potent than 066ATZ. CX-4945 also has a longer half-life ($t_{1/2}$, ~5 h) in mice than 066ATZ. Therefore, the dose (5 mg/kg) chosen for this experiment is expected to produce a greater degree of CK2α/α' inhibition than that achieved by 066ATZ (Fig. 4A).

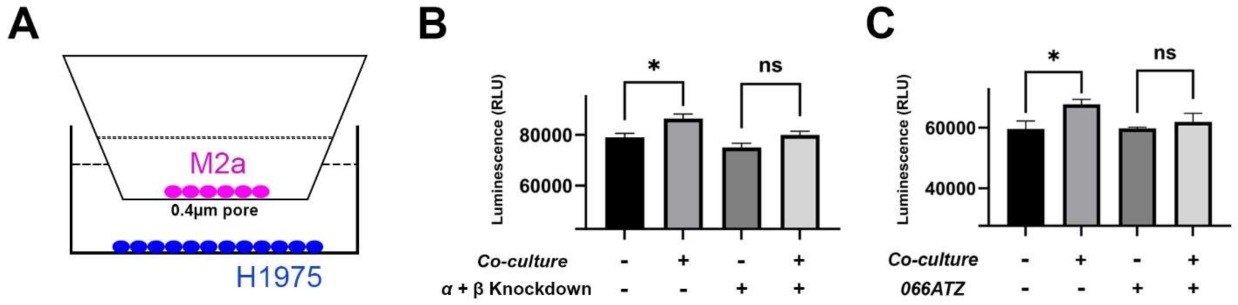

Figure S3. PIP4K2A/2B inactivation impairs the growth-promoting effect of M2a macrophages. (**A**) A schematic illustration of the co-culture experiment with M2a macrophages and H1975 cells. Briefly, 20,000 THP-1 cells were seeded into 24-well Transwell inserts with 0.4 μm membranes (Corning, 353095) and differentiated/polarized into M2a macrophages. The macrophages were then washed once with culture medium and treated with either DMSO or 20 μM 066ATZ for 1 day. For inducible PIP4K2A/2B knockdown, macrophages were treated with 200 ng/mL doxycycline for 5 days. Separately, 20,000 H1975 cells were seeded into 24-well plates and, after 24 hours, washed once with 1× DPBS and serum-free RPMI-1640, then incubated in 500 μL serum-free RPMI-1640. Transwell inserts containing M2a macrophages were placed onto the H1975 cultures for a 3-day co-culture period. H1975 cell number was then determined using CellTiter-Glo®. (**B**) Co-culture with M2a macrophages significantly enhanced H1975 cell growth, whereas knockdown of PIP4K2A/2B in M2a macrophages abolished their growth-promoting effect (*, $p < 0.05$; ns, not significant). Data are presented as mean ± SEM (n=4). Statistical analysis was performed using a two-tailed unpaired t-test with Welch's correction. (**C**) Treatment with 066ATZ likewise eliminated the growth-promoting effect of M2a macrophages (*, $p < 0.05$; ns, not significant). Data are presented as mean ± SEM (n=4). Statistical analysis was performed using a two-tailed unpaired t-test with Welch's correction.

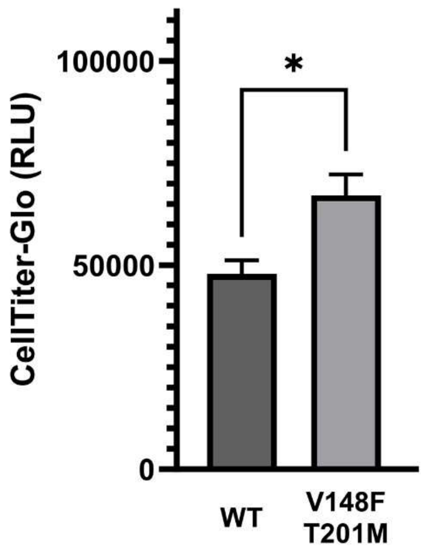

Figure S4. Expression of a refractory PIP4K2A mutant rescues M2a macrophages' growth promoting activity. A THP-1 cell line stably expressing the drug-refractory double mutant V148F/T201M was generated, then differentiated and polarized into the M2a state alongside the parental wildtype (WT) line. After treatment with 20 μM 066ATZ, the conditioned media were collected and added to cultured H1975 cells. After 72 hours, cell numbers were determined using the CellTiter-Glo® assay (*, $p<0.01$). Data are presented as mean ± SEM (n=6). P values were calculated using Student's t test in GraphPad Prism 10.0 (GraphPad Software, San Diego, CA).

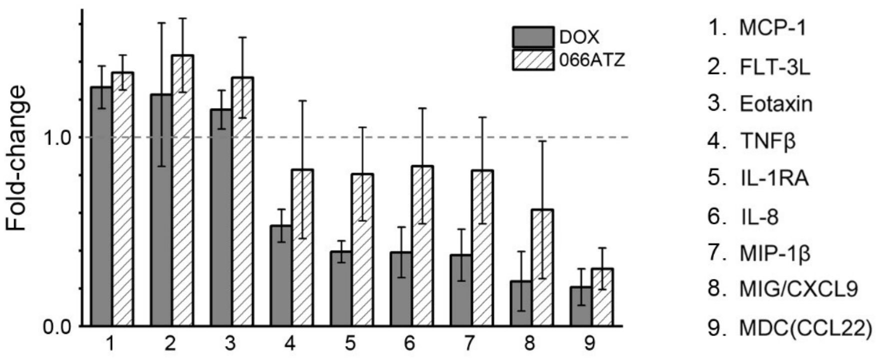

Figure S5. Multiplexed ELISA of the conditioned media from cultured M2a THP-1 macrophages. After treatment with either DMSO (control), DOX (200 ng/ml) or 066ATZ (20 μM), supernatants from three separate wells of cultured cells (75 μl each) were collected and shipped to EVE Technologies Corporation (Calgary, Canada) for Human Panel A 48-Plex Discovery Assay® (HD48A). Among the 48 cytokines/chemokines examined [sCD40L, EGF, Eotaxin, FGF-2, Flt-3 ligand, Fractalkine, G-CSF, GM-CSF, GROα, IFNα2, IFNγ, IL-1α, IL-1β, IL-1ra, IL-2, IL-3, IL-4, IL-5, IL-6, IL-7, IL-8, IL-9, IL-10, IL-12p40, IL-12p70, IL-13, IL-15, IL-17A, IL-17E/IL-25, IL-17F, IL-18, IL-22, IL-27, IP-10, MCP-1, MCP-3, M-CSF, MDC (CCL22), MIG, MIP-1α, MIP-1β, PDGF-AA, PDGF-AB/BB, RANTES, TGFα, TNFα, TNFβ, VEGF-A], 21 were affected by PIP4K2A/2B knockdown, although the differences did not reach statistical significance. Among these, 9 were similarly altered by 066ATZ treatment as shown. The y-axis (fold-change) represents the ratio of the average cytokine concentration in the treatment group over DMSO control. Data are presented as mean ± SEM (n=3). Both DOX and 066ATZ treatments influenced MDC (macrophage-derived chemokine, or CCL22) level the most, but the differences are not statistically significant primarily because the DMSO group has a large SEM (Standard Error of the Mean). It remains uncertain at this moment which of the affected factors contribute to altered tumor cell growth. Nevertheless, the reduction of MDC is of great interest because this chemokine recruits regulatory T cells (Tregs) and plays a role in creating the immunosuppressive tumor microenvironment.

References

1. Siddiqui-Jain, A., Drygin, D., Streiner, N., Chua, P., Pierre, F., O'Brien, S.E., Bliesath, J., Omori, M., Huser, N., Ho, C., et al. (2010). CX-4945, an Orally Bioavailable Selective Inhibitor of Protein Kinase CK2, Inhibits Prosurvival and Angiogenic Signaling and Exhibits Antitumor Efficacy. Cancer research *70*, 10288-10298. 10.1158/0008-5472.Can-10-1893.
2. Pierre, F., Chua, P.C., O'Brien, S.E., Siddiqui-Jain, A., Bourbon, P., Haddach, M., Michaux, J., Nagasawa, J., Schwaebe, M.K., Stefan, E., et al. (2011). Discovery and SAR of 5-(3-Chlorophenylamino)benzo[c][2,6]naphthyridine-8-carboxylic Acid (CX-4945), the First Clinical Stage Inhibitor of Protein Kinase CK2 for the Treatment of Cancer. Journal of medicinal chemistry *54*, 635-654. 10.1021/jm101251q.
